# P-1770. Antimicrobial Stewardship: Usage of AWaRe Classification for justification of antibiotic therapy in a tertiary care hospital in South India

**DOI:** 10.1093/ofid/ofae631.1933

**Published:** 2025-01-29

**Authors:** Suhail Hassan, M Roshni, Rithik Dharan, Sathya Narayanan

**Affiliations:** Tamilnadu Dr. MGR University, Chennai, Tamil Nadu, India; Dr.MGR medical University, Chennai, Tamil Nadu, India; The Tamil Nadu Dr. MGR Medical University, Chennai, Tamil Nadu, India; Tamilnadu Dr.MGR University, Chennai, Tamil Nadu, India

## Abstract

**Background:**

Antibiotic resistance, a consequence of the overuse and inappropriate use of antibiotics, poses a significant global health threat, endangering our ability to effectively treat bacterial infection. Utilizing the WHO,s categorization of antibiotics into Access, Watch, and Reserve (AWaRe) for justification provides a structured approach for assessing antimicrobial use, thereby facilitating the identification of areas for improvement and the implementation of effective antimicrobial stewardship programs (AMS).

**Methods:**

This Prospective Observational study was conducted in a Tertiary care hospital in South India in the month of **APRIL 2024** using a tool containing patient’s demographic data, drug chart , indications, culture reports and justification column.

**Results:**

Out of 1046 patients 246 patient were receiving antibiotics in which the study revealed that a majority of antibiotics fell into the **WATCH (63.4%)**, followed by **ACCESS (33.3%)** and **RESERVE (3.25%)** categories. Among the antibiotics prescribed, Ceftriaxone (28.1%), Cefuroxime (24.8%) and Piperacillin-Tazobactam (22.4%) were most frequently used. Conversely, Fosfomycin, Tigecycline, and Linezolid were prescribed less frequently, each constituting less than 1% of prescriptions. Analysis showed that **164 patients (66.6%)** received antibiotics without justification, while **84 patients (33.4%)** received justified therapy. Regarding patient referral, 26.3% were referred with an ID reference, while the majority (74.6%) were not. Indications for antibiotic use varied, with empirical treatment being the most common (30.5%), followed by prophylaxis (48.9%), and definitive treatment (16.3%). A smaller percentage of cases transitioned from empirical to definitive treatment (6.2%).

**Conclusion:**

In summary, the majority of prescriptions fell under the WATCH category (63.4%), with common antibiotics like Ceftriaxone and Cefuroxime being frequently prescribed. A significant portion (66.6%) of prescriptions lacked justification, highlighting the need for improved antimicrobial stewardship. The low rate of infectious disease referrals suggests potential gaps in consultation. Targeted interventions are necessary to rationalize prescribing practices and address the threat of antimicrobial resistance.

**Disclosures:**

**All Authors**: No reported disclosures

